# Evaluation of a Method for Nitrotyrosine Site Identification and Relative Quantitation Using a Stable Isotope-Labeled Nitrated Spike-In Standard and High Resolution Fourier Transform MS and MS/MS Analysis

**DOI:** 10.3390/ijms15046265

**Published:** 2014-04-14

**Authors:** Kent W. Seeley, Alison R. Fertig, Craig P. Dufresne, Joao P. C. Pinho, Stanley M. Stevens

**Affiliations:** 1 Department of Cell Biology, Microbiology, and Molecular Biology, University of South Florida, 4202 E. Fowler Ave., Tampa, FL 33620, USA; E-Mails: afertig@mail.usf.edu (A.R.F.); costapinho@mail.usf.edu (J.P.C.P.); 2 Training Institute, Thermo Fisher Scientific, 1400 Northpoint Parkway, Ste 10., West Palm Beach, FL 33407, USA; E-Mail: craig.dufresne@thermofisher.com

**Keywords:** nitration, oxidative stress, immonium ion, high resolution mass spectrometry, liquid chromatography (LC)-MS/MS, FTMS

## Abstract

The overproduction of reactive oxygen and nitrogen species (ROS and RNS) can have deleterious effects in the cell, including structural and possible activity-altering modifications to proteins. Peroxynitrite is one such RNS that can result in a specific protein modification, nitration of tyrosine residues to form nitrotyrosine, and to date, the identification of nitrotyrosine sites in proteins continues to be a major analytical challenge. We have developed a method by which ^15^N-labeled nitrotyrosine groups are generated on peptide or protein standards using stable isotope-labeled peroxynitrite (O^15^NOO^−^), and the resulting standard is mixed with representative samples in which nitrotyrosine formation is to be measured by mass spectrometry (MS). Nitropeptide MS/MS spectra are filtered using high mass accuracy Fourier transform MS (FTMS) detection of the nitrotyrosine immonium ion. Given that the nitropeptide pair is co-isolated for MS/MS fragmentation, the nitrotyrosine immonium ions (at *m*/*z* = 181 or 182) can be used for relative quantitation with negligible isotopic interference at a mass resolution of greater than 50,000 (FWHM, full width at half-maximum). Furthermore, the standard potentially allows for the increased signal of nitrotyrosine-containing peptides, thus facilitating selection for MS/MS in a data-dependent mode of acquisition. We have evaluated the methodology in terms of nitrotyrosine site identification and relative quantitation using nitrated peptide and protein standards.

## 1. Introduction

Protein modifications induced by oxidative or nitrative stress have been implicated in the pathophysiological mechanisms underlying the progression of certain diseases [1–9]. Protein tyrosine nitration (PTN) is characterized by covalent addition of a nitro- group (–NO_2_) to the ortho-position of the phenolic ring of protein-bound tyrosine residues. PTN alters the physicochemical properties of the modified tyrosine residue and can confer changes to the local chemical environment and protein structure, resulting in the alteration of protein function, turnover rates, protein-protein interactions and cell signaling [10–12]. A major obstacle to mass spectrometric PTN identification is the sensitivity required to identify specific nitration sites from a proteome-wide analysis, since nitrotyrosine occurs at abundances ~five orders of magnitude lower than unmodified tyrosine residues [13,14]. Accordingly, if signal-to-noise and detection specificity can be enhanced to facilitate mass spectrometry (MS)-based detection of nitrotyrosine-containing peptides, nitration site identification from complex biological matrices could be feasible using standard MS data acquisition methods, providing an alternative strategy that complements chemical derivatization and enrichment techniques that have been employed prior to MS-based analysis of low-abundance post-translational modifications (PTM), such as PTN [15–17].

Among the MS-based methods to facilitate protein PTM identification [18,19], precursor ion scanning on a triple quadrupole mass spectrometer or hybrid instrumentation with similar capabilities has been used to detect peptide masses that generate MS/MS fragment ions specific for a particular PTM [20]. One such approach entails using the PTM-modified immonium ion as a diagnostic marker for the PTM. Immonium ions are internal fragment ions specific to each of the twenty common amino acid residues that are formed by a double backbone cleavage characteristic of both y- and a-type ion production, resulting in the general structure, H_2_N^+^ = CH–R [21,22]. Most immonium ions appear at low frequency; however, certain immonium ions representing histidine, isoleucine, leucine, phenylalanine, proline, tryptophan and tyrosine can generate stronger signals depending on MS/MS dissociation characteristics [22–24]. Given the stronger signal observed for the tyrosine immonium ion, it has been shown to be a useful marker for the detection of tyrosine-targeted modifications, such as phosphorylation or nitration using precursor ion scanning [20,24,25]. Some limitations of this approach are the potential isobaric interference of the nitrotyrosine immonium ion by other fragment ions at low mass resolution and the lack of sensitivity and specificity required to detect endogenous tyrosine nitration sites using precursor ion scanning on a chromatographic timescale.

In addition to identification, relative quantitation of PTN by mass spectrometry using isotope-coded tags has been previously described [26,27], yet evaluation of relative quantitation methods that do not require chemical manipulation of the nitro- group is lacking. Given previous work that utilized the diagnostic immonium ion for nitrotyrosine detection, our attention was drawn to a novel stable isotope labeling by an amino acids in cell culture (SILAC) method for global-scale relative protein quantitation that used isobaric forms of amino acids for protein labeling in different groups [28]. MS analysis yielded peptide precursor ions at the same nominal mass; however, upon MS/MS fragmentation, immonium ions pairs representing the different experimental groups were separated by 1 Da. This immonium ion splitting (ISIS) approach allowed for both sequence and quantitative data using the MS/MS spectra of the labeled peptides. We have developed a stable isotope-labeling and MS-based detection strategy designed to capitalize on the advantages of the previously described methods as a sensitive and quantifiable PTN-specific characterization approach that uses high-resolution Fourier transform MS (FTMS) and MS/MS analysis. Specifically, the unique nitrotyrosine immonium ion can be used as a reporter ion for PTN identification, but can also be used for relative quantitation of nitropeptides from complex protein mixtures. Quantitation can be achieved using the ratios of the immonium ion peak pairs where the relative contributions from proteins reacted with peroxynitrite or endogenously-generated nitroproteins (immonium ion at *m*/*z* 181.0608), and standards reacted with synthetic heavy-labeled peroxynitrite (immonium ion at *m*/*z* 182.0578, used as an internal standard) result in a ratio that is relevant to nitropeptide and, ultimately, nitroprotein abundances. An advantage of using FTMS detection for this method includes ultrahigh mass accuracy filtering of nitropeptide-derived MS/MS spectra to minimize false positive identifications. Additionally, isotopic interference in the nitrotyrosine immonium ion mass region can be significantly minimized at a mass resolution greater than 50,000 (FWHM, full width at half-maximum) in MS/MS mode, a resolution routinely obtainable with FTMS instrumentation, thus allowing for higher relative quantitation accuracy. In this report, we describe the development and assessment of this mass spectrometry-based method for nitrotyrosine identification and relative quantitation using peptide and protein standards for evaluation.

## 2. Results and Discussion

### 2.1. Method Overview

**Figure 1.**
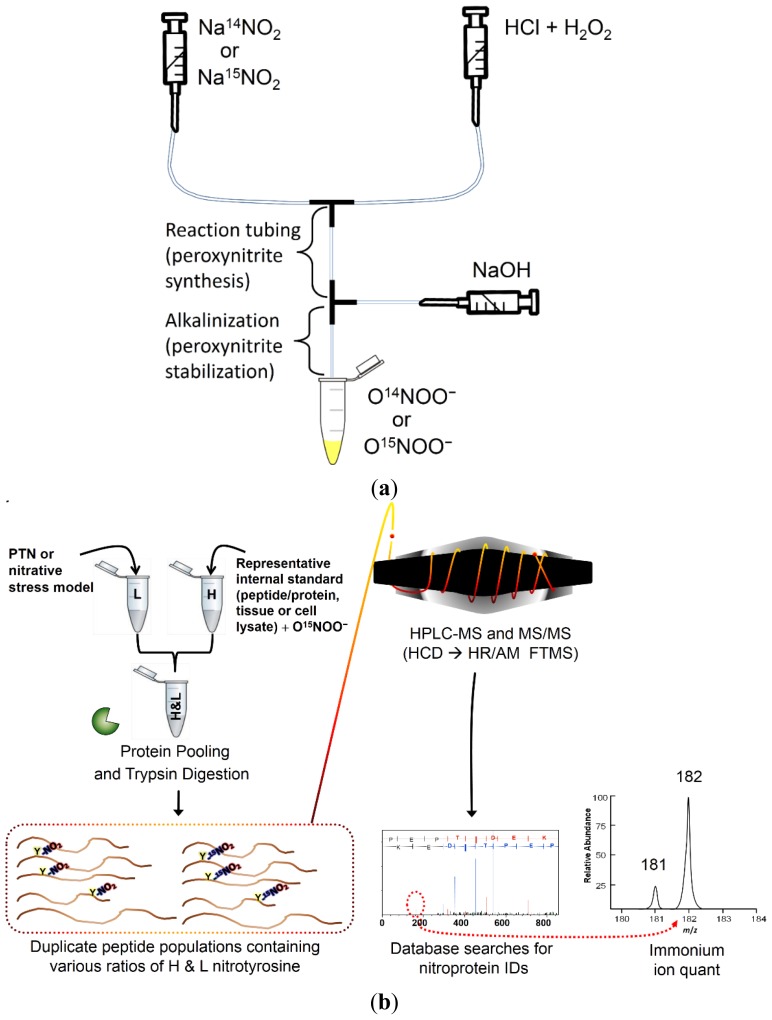
(**a**) Experimental setup used for standard or ^15^N-labeled peroxynitrite synthesis; and (**b**) the proposed workflow for nitration identification and relative quantitation using the stable isotope-labeled nitrated spike-in standard approach. L, light-labeled peroxynitrite; H, heavy-labeled peroxynitrite; PTN, protein tyrosine nitration; HCD, higher-energy collision-induced dissociation; HR, high resolution; AM, accurate mass; FTMS, Fourier transform MS.

The method reported here is expected to facilitate the identification and relative quantitation of PTN in complex protein mixtures. To initiate this process, stable isotope-labeled (heavy) O^15^NOO^−^ was synthesized using a syringe pump platform, as shown in Figure 1a and described further in Section 2.2. The heavy ONOO^−^ can then be reacted with protein standards, cell lysates or tissue protein extracts that are representative of the sample in which nitration will be measured (*i.e.*, biological system in which nitrative stress occurs). This protein mixture containing heavy-labeled nitration serves as an internal standard that is then spiked into the sample where endogenous nitration will be identified and quantified (Figure 1b). Since nitropeptide pairs are differentiated by 1 Da, a standard isolation window for precursor ion selection can be specified for MS/MS, which is lower than the *m*/*z* 10 window described earlier for multiplexed MS/MS quantitation using precursors with a Δm of three [29]. Nitrotyrosine-containing y- or b-ion series are expected to exhibit doublet peaks with a Δm of 1 Da, which could be quantified by a method similar to the one described by Zhang and Neubert [29], using ratios of MS/MS fragment ions for quantitation. Preferably, our method will quantify based on the ratio of peak intensities from the heavy and light nitrotyrosine immonium reporter ion pairs.

There are several advantages to using ratios of nitrotyrosine immonium ions for PTN identification and quantitation with FTMS detection. First, since the nitroproteome should be modest in size, manual screening for nitrotyrosine immonium ion pairs is feasible through the reconstruction of ion chromatograms to detect MS/MS spectra containing ions within the *m*/*z* range of 181–182 at high resolution. Moreover, our focus is specific to a single isotope pair, negating the necessity for the development of a complex search algorithm to pre-qualify peak pairs for quantitation. Finally, the low-mass immonium reporter ions are specific to nitrotyrosine in that they neither overlap with any of the other twenty immonium ions, and high resolution FTMS detection minimizes overlap with other potential isobaric MS/MS fragment ions.

### 2.2. Stable Isotope-Labeled Peroxynitrite Synthesis

Employing a scaled-down version of a previously-described method [30], we synthesized 30-mL volumes using a syringe pump system to combine NaNO_2_ or Na^15^NO_2_ and acidified H_2_O_2_ in order to produce either light or heavy ONOO^−^ (Figure 1a). Due to ONOO^−^ instability at low pH, concentrated NaOH was added post-reaction from a separate syringe. The product was collected on ice and either stored at −80 °C or assayed for concentration at 302 nm prior to use.

Because our syringe capacity was one-third the volume of the syringes used in the large-scale synthesis [30], optimization steps were necessary to maximize final product yields. After several adjustments to reagent concentrations, tube lengths and storage buffer concentrations, we determined that the following parameters produced the maximum ONOO^−^ yield: 240 mM HCl, 530 mM H_2_O_2_, 1.27 M NaNO_2_ or Na^15^NO_2_, a 3-cm reaction tube length and a 300-mM final NaOH storage buffer concentration. The syringe pump was programmed for the maximum flow rate for all reactions. The limiting factor for reaction yield was likely to have been syringe size, as the maximum flow rate for 100 mL syringes is 415.9 mL/h compared to 862.8 mL/h for 30-mL syringes and 2376 mL/h for 140 mL-syringes. Theoretically, the system described could produce >100 mM ONOO^−^ using 140-mL syringes at full-speed, which is close to previously reported yields [30]. Nevertheless, the yield maximum for ONOO^−^ (24 and 22 mM for heavy and light ONOO^−^, respectively) was expected to be sufficient for our purposes. To the best of our knowledge, this study represents the first instance where stable isotope-labeled peroxynitrite has been used for nitrotyrosine-specific isotope coding for global-scale analysis of protein nitration.

### 2.3. Angiotensin I Peptide Testing

Light or heavy synthetic ONOO^−^ at various concentrations was reacted with 100 μM angiotensin I (DRVYIHPFHL) to validate its reactivity when diluted from the stock solution containing a fairly high concentration of NaOH and also to determine the signal strength of the nitrotyrosine immonium ions from a short peptide using either collision-induced dissociation (CID) or higher-energy collision-induced dissociation (HCD). Peroxynitrite concentrations >800 μM yielded high pH values (≥10) because of the minimal dilution of the OH^−^ used to stabilize ONOO^−^, thus possibly retarding its pH-dependent reactivity with the angiotensin I test peptide. Reactions performed at 400 μM or lower of ONOO^−^ provided a pH range (~8) for efficient nitration to occur.

**Figure 2.**
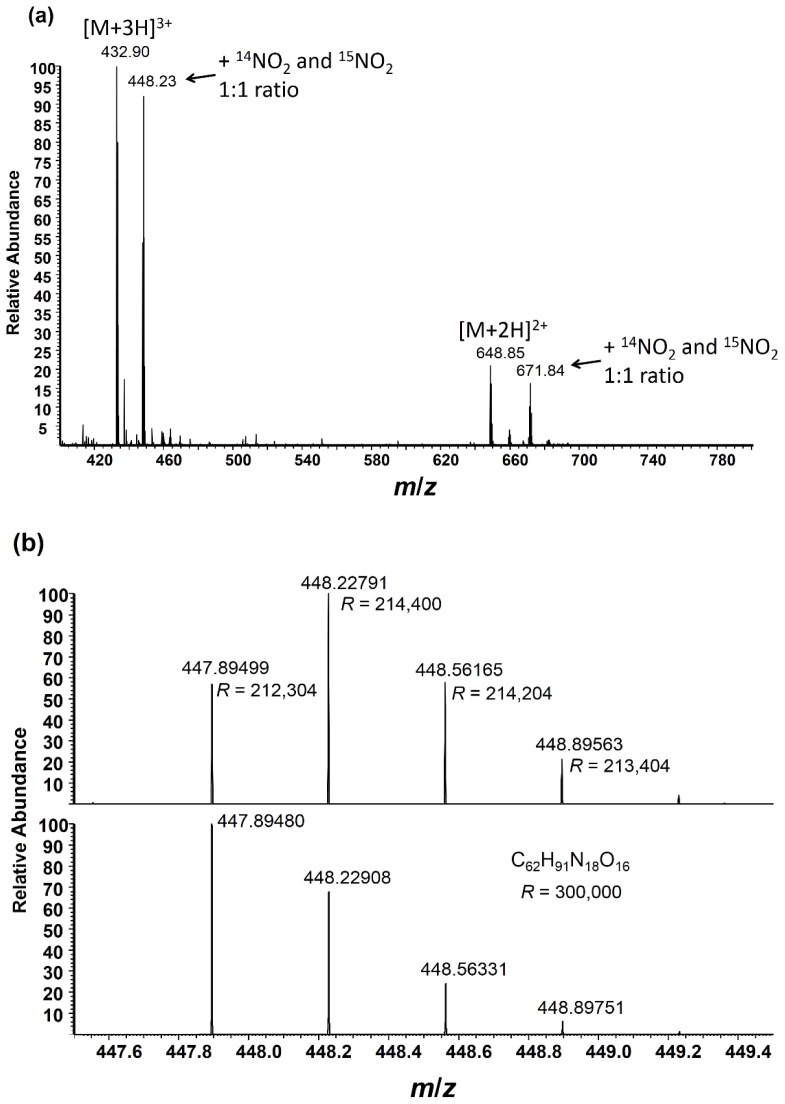

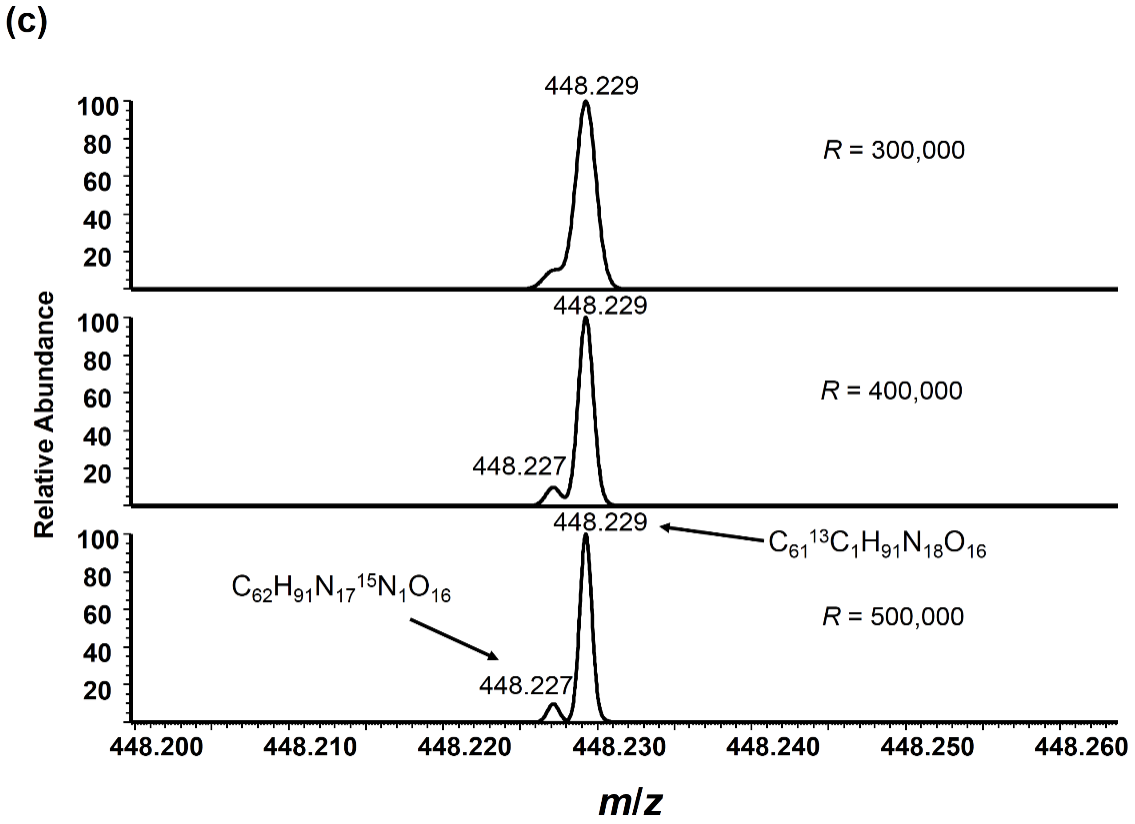
(**a**) Full-scan FTMS (Fourier transform mass spectrum) of a 1:1 mixture of light:heavy nitrated angiotensin I, DRVYIHPFHL; (**b**) Expanded region at *m*/*z* 448 showing the isotope cluster containing a 1:1 mixture of light:heavy nitrated angiotensin I (**top**) and an isotope simulation of nitrated (light only) angiotensin (**bottom**); and (**c**) separation of the heavy (^15^N) nitrated angiotensin I peak from the second isotope peak of light nitrated angiotensin I at three different mass resolution values in a full scan mass spectrum. The “*R*” value indicates the resolution of the acquisition.

Angiotensin I reacted with 200 μM of heavy ONOO^−^ was used as an internal standard by pooling with various dilutions of angiotensin I reacted with 200 μM of light ONOO^−^ (1:1, 0.5:1, 0.25:1). Figure 2a shows the high nitration efficiency obtained at this ONOO^−^ concentration where the pooled heavy and light nitrated angiotensin I signal (for the 1:1 ratio) was almost the same level as the unmodified angiotensin I upon analysis by direct infusion (ESI, electrospray ionization) on a Fourier transform mass spectrometer (Orbitrap Elite™, Thermo Scientific, San Jose, CA, USA). The monoisotopic peak for the +3 charge state of nitrated angiotensin I was found at *m*/*z* 447.89 and the heavy isotope (containing –^15^NO_2_ and the second isotope peak representing ^13^C from “light” nitrated angiotensin I) at *m*/*z* 448.23 (top view in Figure 2b). The contribution of the second isotope peak from the light nitropeptide can be observed in the isotope simulation based on the elemental composition of nitrated (light) angiotensin I alone (bottom view in Figure 2b). A mass resolution of >400,000 (FWHM) at *m*/*z* 448 would be required to separate the heavy monoisotopic peak from the second isotope (^13^C) peak of the light peptide, as shown in Figure 2c, indicating that relative quantitation in full-scan MS could be performed post-sequence identification to determine the elemental composition and corresponding contribution of the second isotope peak from the light nitropeptide prior to ratio calculation, if lower mass resolution instruments are employed. Alternatively, ultrahigh mass resolution detection of nitrated peptide pairs would facilitate relative quantitation in full MS scans, as recently demonstrated by the neutron-encoded SILAC approach in which multiplexed relative quantitation is performed on peptide peaks with small mass differences (mDa range) [31]; however, current commercial instruments are just below the mass resolution needed for the method reported here without compromising data acquisition speeds for high-throughput MS/MS-based peptide sequencing. Full-scan MS quantitation would also depend on the magnitude of natural ^15^N interference, which is generally low, but is specific to the peptide sequence.

**Figure 3.**
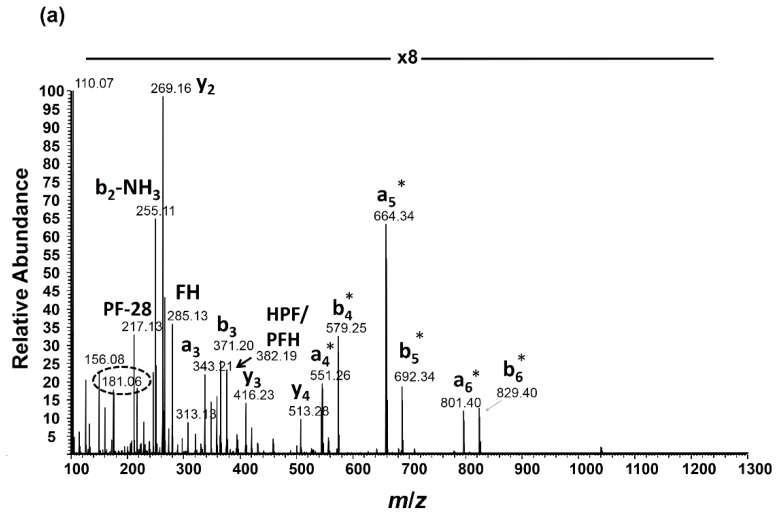

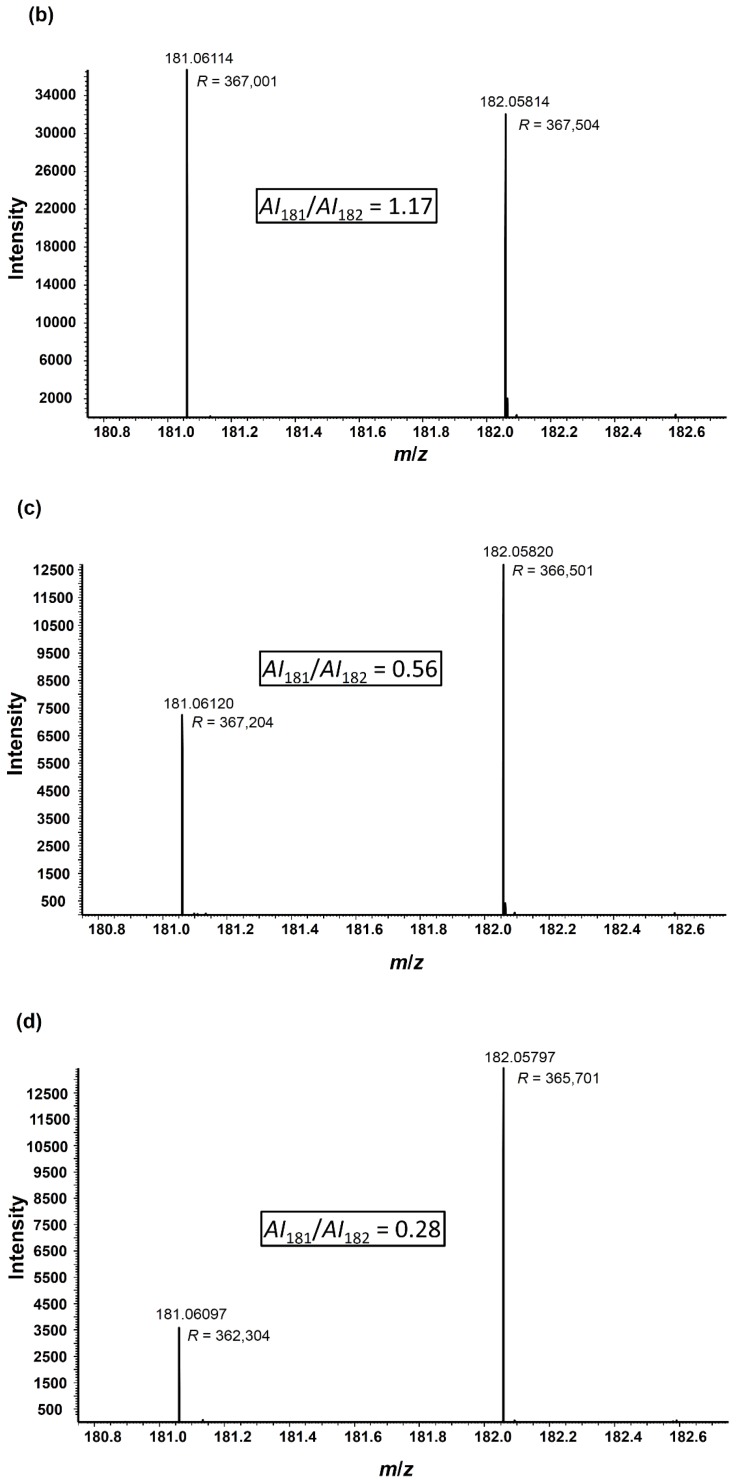

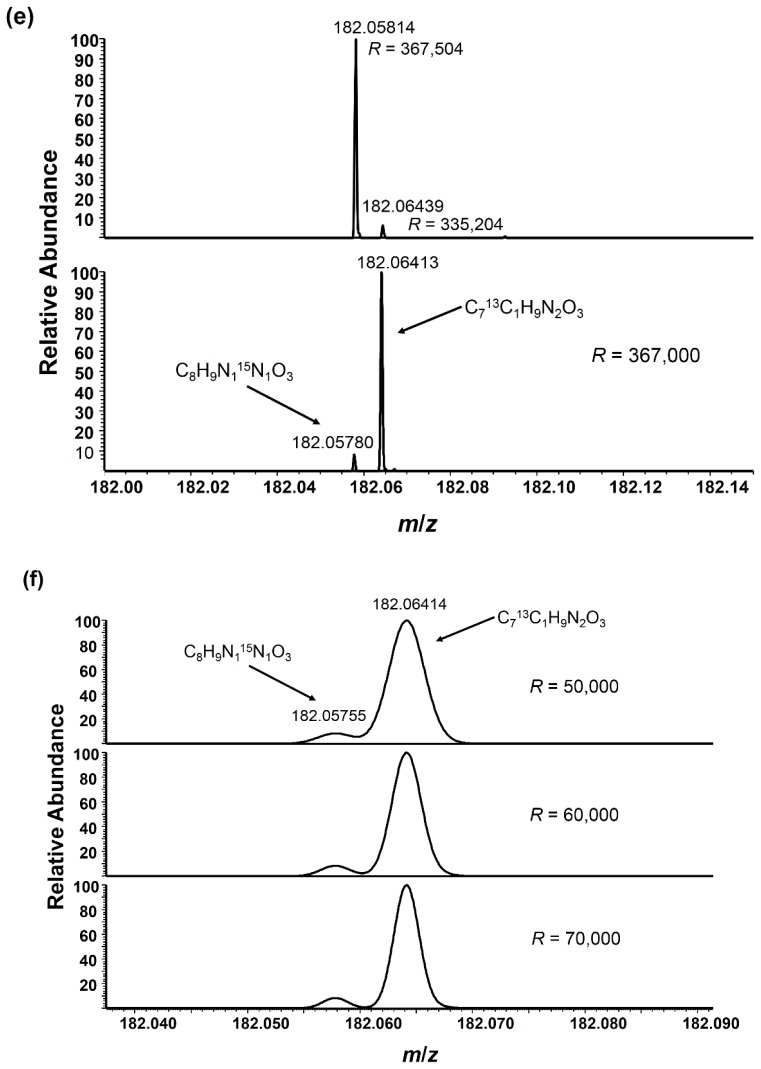
(**a**) Higher-energy collision-induced dissociation (HCD) MS/MS spectrum of nitrated angiotensin I (1:1 (light:heavy) mixture); peak intensities were magnified 8-fold in this *m*/*z* range; (**b**) Nitrotyrosine immonium ions from the 1:1 (light:heavy); (**c**) 0.5:1 and (**d**) 0.25:1 mixture (*AI* = absolute intensity); (**e**) Expanded region at *m*/*z* 182 from the 1:1 mixture (**top panel**) and isotope simulation of the light nitrotyrosine immonium ion expanded at *m*/*z* 182 showing that the mass resolution used in the FTMS analysis can separate this isotopic interference from the naturally occurring ^13^C isotope in the second peak of the unlabeled 3NT immonium ion (**bottom panel**); and (**f**) Isotope simulation of the light nitrotyrosine immonium ion focusing on isotope peaks at *m*/*z* 182. Separation of isotope interference occurs at mass resolution values ≥50,000. * indicates fragment ions that contain the nitrotyrosine modification. The “*R*” value indicates the resolution of the acquisition.

MS/MS analysis using either HCD or CID was performed on pooled samples containing various ratios of light:heavy nitrated angiotensin I. The MS/MS spectrum of the light and heavy nitropeptide pair (1:1 ratio) co-isolated for HCD fragmentation is shown in Figure 3a. Using HCD in the hybrid Orbitrap instrument employed in this study, quadrupole-like fragmentation (22–30 eV) was obtained, allowing for enhanced production of the nitrotyrosine immonium ions. Based on the HCD analysis of the +2 and +3 charge states of angiotensin I, the higher charge state also allowed for enhanced production of the nitrotyrosine immonium ions. HCD was also preferred over CID given the issue associated with limited low mass ion detection (low mass cutoff (LMCO) of 28% precursor mass at *q*_z_ = 0.25) when carrying out CID MS/MS fragmentation in the linear ion trap prior to ion transmission to the Orbitrap for high resolution fragment ion detection. The *q*_z_ value can be manually adjusted from the default value of 0.25 in order to modify the LMCO; however, this option was not pursued in this study. The nitrotyrosine immonium ions derived from light:heavy nitrated angiotensin I allowed for relative quantitation of the nitropeptide (Figure 3b–d) and calculation of accurate ratio values after normalization. The ratio range used reflects that anticipated for samples obtained from biological systems of nitrative stress where the heavy nitrated internal standard will presumably be at a higher level as a result of *in vitro* peroxynitrite reactions. Additionally, the internal standard will be spiked in at the same concentration across multiple samples in order to normalize during the relative quantitation procedure [*i.e.*, (nitropeptide Condition 1/internal standard)/(nitropeptide Condition 2/internal standard)]. The normalized ratio values reported here used the ratio value calculated for the 1:1 ratio as the reference point for normalization [(nitropeptide Condition 2/internal standard) in the previous equation].

High mass resolution detection is required to minimize isotopic interference that arises from the second isotope (^13^C) peak of the light nitrotyrosine immonium ion with the heavy (^15^N) nitrotyrosine peak. Figure 3e (top view) is an expansion of this region at *m*/*z* 182 showing the accurate mass detection of the heavy nitrotyrosine immonium ion peak (from 1:1 ratio in Figure 3b) with sufficient separation from the second isotope peak of the light nitrotyrosine immonium ion at the maximum mass resolution setting of the FT mass spectrometer used in this analysis (240,000 at *m*/*z* 400 corresponding to 748 ms transient acquisition). The bottom view is an isotope simulation based on the elemental composition of the light nitrotyrosine immonium ion showing the relative amount of the second isotope peak at *m*/*z* 182 within the isotope cluster. It is important to note that there is some ^15^N contribution to the heavy nitrotyrosine immonium peak from the unlabeled nitrotyrosine immonium; however, this amount is small (<1% of the monoisotopic peak height of the light nitrotyrosine immonium ion) and can be considered negligible given the error associated with the ratio measurement (reported in Section 2.4 and Table 1). The mass resolution setting employed for MS/MS analysis of nitrated angiotensin I was ample in terms of minimizing isotopic interference at *m*/*z* 182, but the mass resolution of ≥50,000 was considered sufficient, as determined by isotope simulations at various mass resolution values (Figure 3f).

### 2.4. Protein Standard Testing

A protein standard, bovine serum albumin (BSA), was reacted with 200 μM light or heavy ONOO^−^, and relative quantitation of the nitration sites was evaluated by determining the ratio of nitrotyrosine immonium ions detected from differential dilutions of light ONOO^−^-treated BSA combined with heavy ONOO^−^-treated BSA that was used as an internal standard. The ratios generated were 1:1, 0.25:1 and 0.10:1 (light:heavy nitro-BSA) and added to a more complex matrix, a cell lysate digest of immortalized rat microglia recently characterized by our lab [32,33]. Mass spectrometric data files were searched against the Uniprot rat database and a second database consisting of known contaminants including BSA using MaxQuant (Max Planck Institute of Biochemistry, Martinsried, Germany) [34]. Additionally, manual accurate mass reconstruction of ion chromatograms to identify MS/MS spectra containing the heavy nitrotyrosine immonium ion was used to confirm database search results, but also to detect other potential nitrotyrosine sites not identified by MaxQuant. Database searching using MaxQuant identified 1121 unique protein groups (excluding decoy sequences) across all raw data files. Several peptides from BSA were identified, which included one nitropeptide, Y*ICDNQDTISSK (*m*/*z* 745.3), from the 0.25:1 mixture. For nitropeptides in which nitrotyrosine is the *N*-terminal residue, the a_1_ ion is formed, giving rise to the same *m*/*z* value as the internal immonium ion. To be inclusive, we refer to the fragment ions at *m*/*z* 181 or 182 as nitrotyrosine reporter ions in the subsequent sections of this report.

**Figure 4.**
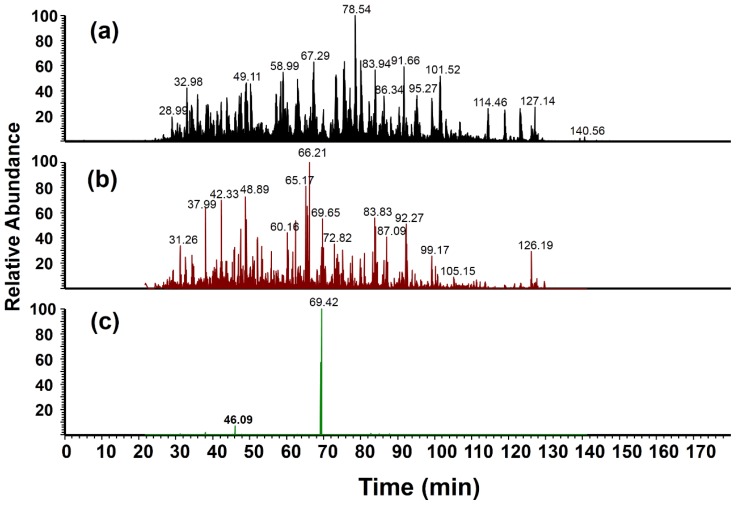
(**a**) Base peak ion chromatogram obtained from liquid chromatography (LC)-MS/MS analysis of a microglial cell lysate digest with nitro-BSA mixture (0.25:1 (light:heavy)) added; (**b**) Reconstructed ion chromatogram showing MS/MS spectra that contain the *m*/*z* 182 peak with a detection mass tolerance of ±1.0 Da (*m*/*z* 181.0–183.0); and (**c**) Reconstructed ion chromatogram showing MS/MS spectra with high mass accuracy detection of the *m*/*z* 182 peak (*m*/*z* 182.056–182.058).

The benefit of high mass accuracy and resolution FTMS detection is demonstrated in Figure 4. For example, Figure 4b is a reconstructed ion chromatogram profile showing MS/MS spectra with a signal derived from *m*/*z* 182 within a mass window of ±1 Da. The profile obtained is similar to that of the base peak ion chromatogram of the cell lysate digest (Figure 4a), indicating that lower mass resolution instruments are limited in terms of specificity for nitropeptide detection. Upon accurate mass reconstruction in which the mass window for detection was decreased to ±0.001 Da, the complexity of the chromatogram was significantly reduced, where two major peaks at 46 and 69 min (high-performance liquid chromatography (HPLC) retention time) were observed, as shown in Figure 4c. These two major peaks correspond to two unique nitropeptides detected from BSA. One of the nitropeptides was identified previously by database searching (Y*ICDNQDTISSK is the peak at 45–48 min range), while the other was identified by *de novo* sequencing using the MS/MS spectrum at 69 min. The sequence of the second peptide was determined to be Y*LYEIAR (*m*/*z* 487.2) and was reproducibly identified across all samples after filtering MS/MS spectra based on the requirement of high mass accuracy detection of the heavy nitrotyrosine reporter ion. It is unclear why this particular nitropeptide was not identified by MaxQuant; however, this result provides some evidence to support the use of multiple search algorithms to enhance peptide identifications. This nitrated peptide has also been identified in other studies where similar modification site selectivity has been observed [15,20]. Our group has further described the influence of local chemical environment on tyrosine nitration selectivity [35].

**Figure 5.**
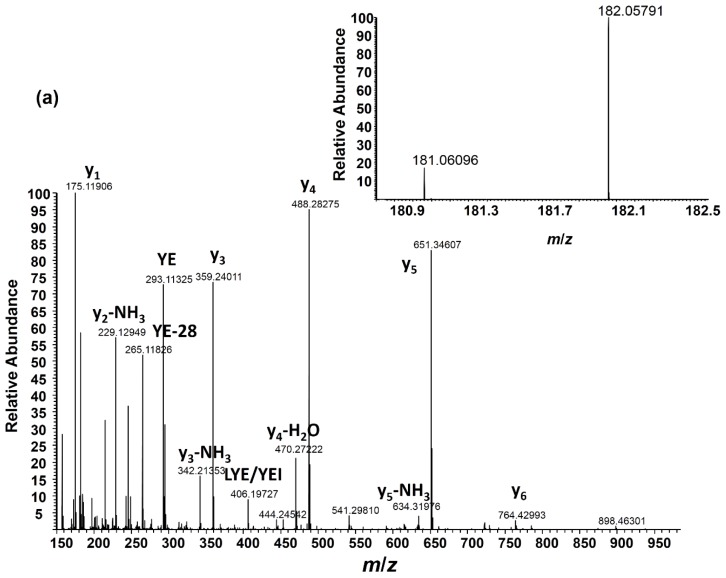

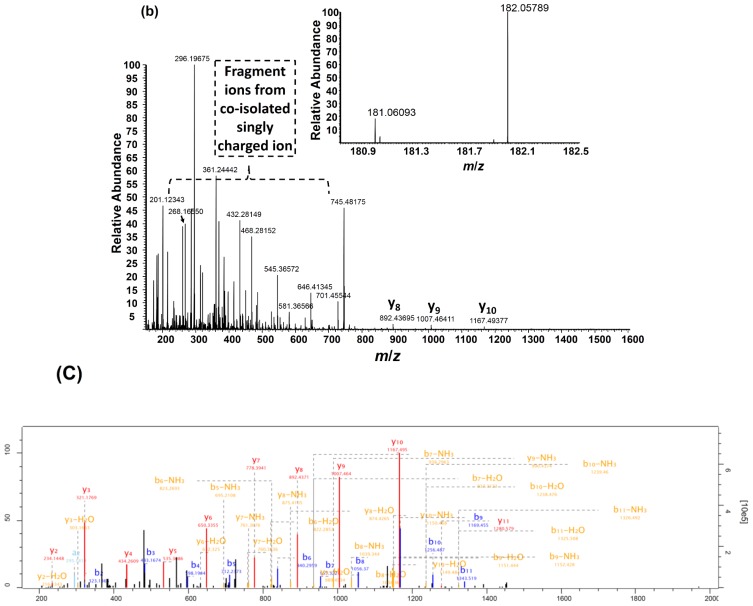
(**a**) HCD MS/MS spectrum of *m*/*z* 487.2 (+2 charge state), the nitropeptide, Y*LYEIAR, from BSA; (**b**) HCD MS/MS spectrum of *m*/*z* 745.5 (+2 charge state), the nitropeptide, Y*IC^#^DNQDTISSK, from BSA. Insets in both spectra show the expanded region containing the light and heavy nitrotyrosine reporter ion pair; and (**c**) Collision-induced dissociation CID MS/MS spectrum of *m*/*z* 745.5 used in the identification of Y*IC^#^DNQDTISSK by MaxQuant. * and ^#^ indicate nitration and carbamidomethylation, respectively.

In addition to nitropeptide identification, relative quantitation of nitropeptide amounts from the different ratio mixtures was successful within the ratio range evaluated. The MS/MS spectra of Y*LYEIAR and Y*ICDNQDTISSK from the 0.25:1 ratio samples and fragmented by HCD are shown in Figure 5a,b, respectively. Based on previously described guidelines for nitrotyrosine identification by MS/MS [36], the nitropeptide, Y*LYEIAR, was confirmed successfully. The HCD spectrum of Y*ICDNQDTISSK was complicated by the co-isolation of a more abundant isobaric singly charged species at that particular chromatographic time point. Consequently, masses lower than *m*/*z* 745 that could not be annotated were assumed to be associated with this isobaric interference. Nevertheless, high mass accuracy FTMS detection of the precursor ion, higher mass fragment ions (containing consecutive y-ion series) and the nitrotyrosine reporter ion pair in addition to database search identification through a CID MS/MS spectrum at an earlier time point (Figure 5c) allowed for confident identification of this nitropeptide. Upon closer inspection of the nitrotyrosine reporter ion region, the experimentally-derived ratios (intensity of *m*/*z* 181/intensity of *m*/*z* 182), as shown in the insets of Figure 5a,b, are close to the expected value of 0.25. However, given that this technique will be used as shown in Figure 1b, the ratios were normalized to the internal standard, which is determined by the heavy nitropeptide reporter ion signal. As mentioned previously, the BSA nitropeptide, Y*LYEIAR, was consistently identified across all samples, allowing for relative quantitation of this nitropeptide in each ratio mixture. In this case, the normalized ratio accurately reflected the change expected from decreasing the light nitro-BSA amount, as shown in Table 1.

### 2.5. Method Evaluation Summary: Implications in Global-Scale Nitration Analysis

Given the small mass difference (1 Da) generated for the nitropeptide pairs, ultrahigh mass resolution detection in full scan mode would be required to minimize isotopic interference from the unlabeled peptide’s second (^13^C) isotope peak for quantitation. As hybrid FTMS technology continues to improve, we anticipate that full scan mass spectrum-based quantitation could be possible for this technique without significantly limiting peptide identification by parallel MS/MS spectra acquisition, as recently demonstrated in the NeuCode SILAC method [37]. MS/MS analysis, however, does allow for co-isolation of the nitropeptide pair followed by relative quantitation of the nitrotyrosine reporter ions at a mass resolution that is feasible for high-throughput peptide sequencing using LC-MS/MS on the current FT mass spectrometer used in this study. Additionally, there is negligible interference by the ^15^N peak of the unlabeled nitrotyrosine reporter ion based on the elemental composition of this low-mass ion, which might not be the case for full scan MS-based quantitation. It is important to note that in addition to the nitrotyrosine reporter ion pair, fragment ion distribution will also change depending on the location of the nitrotyrosine residue within the peptide sequence, which needs to be considered for database searching or *de novo* sequencing purposes. Although uncommon, peptide sequences with multiple nitrotyrosine residues would require adequate fragment ion coverage to perform relative quantitation of nitration at a site-specific level. In our experience, CID-based fragmentation does not show a neutral loss or possible scrambling of the NO_2_ group [35,36], yet these events should perhaps be considered during data analysis, particularly when HCD/CID parameters have been modified considerably or other dissociation methods are utilized. Extensive characterization of nitropeptide fragmentation by various dissociation methods has been previously reported by other labs [38–40]. Alternatively, multiply nitrated peptides or other nitropeptides that are detected, yet for which relative quantitation is not accomplished, could be used for further targeted validation using complementary approaches (e.g., triple quadrupole MS analysis). Although the method described here is designed for relative quantitation of nitration, absolute quantitation could be performed using initial nitrotyrosine reporter ion results as a guide for targeted quantitative analysis with techniques such as stable-isotope dilution mass spectrometry.

Stable isotope-labeled (^18^O-labeled) peroxide could be used in peroxynitrite synthesis (Figure 1a) to generate a greater mass difference for the peptide pair; however, we were concerned about the isotopic purity of the final product and also that the residual peroxide that remains could generate other labeled (oxygen-based) modifications that would confound data analysis. Nevertheless, there is a major anticipated benefit of the small mass difference for global-scale nitration analysis in that the stable isotope-labeled nitrated spike-in standard would potentially increase the overall signal of the second isotope (^13^C) peak of endogenously nitrated peptides, allowing for data-dependent co-isolation and fragmentation of both species. This advantage would be particularly useful for nitropeptide detection, given that these species are at low-abundance and typically buried within the spectral noise level. Assuming similar chemical selectivity *in vitro* for the nitrated spike-in standard, this approach could minimize and/or complement the use of enrichment or other sample preparation steps prior to MS analysis. Based on the reported success at the peptide and protein standard level, future work will focus on implementation of this approach to assess changes in nitrative stress of relevant biological systems.

## 3. Experimental Section

### 3.1. Peroxynitrite Synthesis

The following items were obtained to build a custom syringe pump-driven chemical synthesis system for standard and stable isotope-labeled peroxynitrite. A NE-1600 six-syringe pump capable of syringe size-dependent infusion rates from 0.452 μL/h to 1451 mL/h was purchased from New Era Pump Systems (Farmingdale, NY, USA). Monoject plastic syringes with Luer tips, a polypropylene Luer connector kit, 3.2 mm inside diameter (ID)/4.8 mm OD Tygon R-3603 tubing (for reagents and NaOH) and 2.4 mm ID/4.0 mm OD Tygon R-3603 tubing (reaction tube) were obtained from Harvard Apparatus (Holliston, MA, USA).

Isotope-labeled and unlabeled peroxynitrite were synthesized from either “heavy” ^15^N-sodium nitrite (Icon isotopes, Summit, NJ, USA) and “light” ^14^N-sodium nitrite, respectively, by reaction with acidified hydrogen peroxide (H_2_O_2_) using a modified version of a previously described method [30]. Briefly, –NO_2_ or –^15^NO_2_ and H_2_O_2_ were delivered to a “T”-junction at a static flow-rate using the six-syringe pump. Volumes of reagents and reaction times were controlled by pump speed, syringe size, tube lengths and tubing inside diameters (IDs).

Reactions were immediately quenched by downstream infusion of 1 M NaOH to a final concentration of 0.3 M (pH 13). Products were collected on ice prior to spectrophotometric concentration assay at the absorbance maximum for ONOO^−^ of 302 nm [41] using a NanoDrop 1000 (Thermo Fisher Scientific, Rockford, IL, USA). The stock solutions were distributed into one-milliliter aliquots and immediately stored at −80 °C to minimize degradation. Assay results were followed by manipulations of reagent concentrations and the reaction tube length to optimize product yields. Syringe size (10 mL) was static, and pump speed was maintained at the maximum flow-rate of 415.9 mL/h for 10-mL syringes. The configuration for this syringe pump system is shown in Figure 1a.

### 3.2. Stable Isotope Labeling Using Synthetic Peroxynitrite

#### 3.2.1. Peptide Standard Reactions

Angiotensin I (Bachem, Torrance, CA, USA) was diluted to 100 μM in PBS and reacted with 200 μM heavy or light ONOO^−^ for 15 min at 37 °C. Reactions were quenched by the addition of formic acid to a final composition of 5%. In a total volume of 100 μL, light and heavy angiotensin I nitration reactions were combined to generate mixtures of 1:1, 0.5:1 and 0.25:1 ratios of light:heavy nitro-angiotensin I. All dilutions were desalted in C18 SPE columns (The Nest Group, Southborough, MA, USA) and eluted in 90:10 acetonitrile:HPLC-grade water (0.1% formic acid). Desalted samples were concentrated under vacuum at 50 °C until dried out completely. Light and heavy nitrated angiotensin I mixtures were resuspended to 100 μM total angiotensin I (assuming 100% recovery) in HPLC-grade 0.1% formic acid in water.

#### 3.2.2. Protein Standard Reactions

Ten milligrams of lyophilized BSA (Thermo Fisher Scientific) were resuspended in 10 mL of PBS. Separate reactions with 500 μL of 1 μg/μL BSA were carried out with either 200 μM heavy or light ONOO^−^. The pH was measured following the addition of the ONOO^−^, which resulted in a final pH of ~8 for both reactions. Reactions were incubated for 15 min at 37 °C. Reactions were then quenched by the addition of formic acid to a final composition of 5%. Nitration reactions were confirmed by running the BSA samples from each reaction on an 8%–16% SDS-PAGE gel (Thermo Fisher Scientific) followed by western analysis with an anti-nitrotyrosine antibody (#321900, Invitrogen, Frederick, MD, USA). Reactions were digested in-solution overnight with trypsin after protein alkylation (cysteine carbamidomethylation) in 6 M urea at room temperature (RT) followed by desalting of the samples using C18 SPE columns (The Nest Group). Samples were concentrated under vacuum at 50 °C until dried out completely. Digested nitro-BSA samples were then resuspended to a final concentration of 1 μg/μL in HPLC-grade water (0.1% formic acid).

Fifty microliters of the heavy ONOO^−^-treated BSA were combined with an equal volume of the light ONOO^−^-treated BSA to produce a 1:1 ratio with a heavy concentration at ~7.5 μM. Light ONOO^−^-treated BSA was separately diluted two-, four- and ten-fold in a total volume of 50 μL. Each dilution was combined with 50 μL of the heavy ONOO^−^-treated BSA reaction to produce three ratios of heavy:light (1:1, 0.25:1, 0.10:1) with the concentration of the heavy ONOO^−^-treated BSA remaining constant in all samples at ~7.5 μM.

Rat highly aggressive proliferating (HAPI) cells were grown to 95% confluency in DMEM media supplemented with 1× penicillin-streptomycin-glutamine and 5% fetal bovine serum. After reaching confluency, cells were washed twice and scraped in ice-cold PBS. Cells were pelleted by centrifugation for 5 min at 500× *g* and then resuspended in 250 μL of 125 mM Tris-HCl, 125 mM dithiothreitol, pH 7.6. Following resuspension, 30% *w*/*v* sodium dodecyl sulfate (SDS) was added to a final concentration of 100 Tris-HCl, 4% *w*/*v* SDS, pH 7.6 with 100 mM dithiothreitol (DTT). Cells were lysed by heating for 5 min at 95 °C and then sonicated. Remaining cellular debris was removed by centrifugation at 16,000× *g* for 5 min, and the supernatant was collected for digest. The protein concentration of the lysate was determined using the Pierce 660 nm Protein Assay with the addition of Ionic Detergent Compatibility Reagent (Thermo Fisher Scientific). Approximately 350 μg of lysate were digested with trypsin on Microcon-30 centrifugal filters (Merck Millipore Ltd., Darmstadt, Germany) using the filter-aided sample preparation (FASP) method [42]. Following digestion, the HAPI cell lysate was desalted, concentrated and resuspended at 1 μg/μL in HPLC 0.1% formic acid in water. Different dilutions of the heavy:light ONOO^−^-treated BSA were spiked into the HAPI cell lysate digest by adding 7.7 μL into 50 μL of the cell lysate suspension to a final concentration of 1 mg/mL cell lysate and 1 pmol/μL of the heavy ONOO^−^-treated BSA.

### 3.3. Liquid Chromatography and Fourier Transform Mass Spectrometry (FTMS) Analyses

#### 3.3.1. Direct Infusion Electrospray Ionization (ESI)-FTMS

Full scans of pooled heavy and light ONOO^−^-treated angiotensin I at different ratios were set to analyze from *m*/*z* 400–800. Samples were delivered via a syringe pump at 3 μL/min in 30% acetonitrile, 70% water with 0.1% formic acid. MS/MS spectra were acquired on the doubly and triply charged nitrated angiotensin I peptide using either CID or HCD on a hybrid linear ion trap-Fourier transform instrument (Orbitrap Elite™, Thermo Scientific). The mass resolution for the MS and MS/MS modes of acquisition was 240,000 at *m*/*z* 400. Normalized CID energy was set to 35%. Normalized HCD collision energy ranged stepwise from 15% to 50% (11–38 eV) to optimize immonium ion production, while maintaining an adequate fragment ion signal across the full *m*/*z* range of the MS/MS spectra.

#### 3.3.2. Liquid Chromatography (LC)-MS/MS Analysis of a Microglial Cell Lysate with Nitrated Bovine Serum Albumin (BSA) Spike-In

Five microliter injections of the heavy and light ONOO^−^-treated BSA digests spiked into a microglial cell lysate were separated by reversed-phase HPLC using an EASY-nLC 1000 (Thermo Fisher Scientific) equipped with an EASY-Spray™ 25 cm × 75 μm ID, C_18_ column (2-μm particle size). Peptides were initially trapped on a 2 cm × 75 μm ID, Acclaim^®^ PepMap™ C_18_ (2-μm particle size) trap column (Thermo Fisher Scientific) loaded in constant pressure mode. The mobile phase flow-rate was 350 nL/min and followed a linear gradient of 2%–25% mobile Phase B over 180 min (mobile Phase A was 0.1% formic acid in water, and B was 0.1% formic acid in acetonitrile) followed by a gradient of 25%–98% mobile Phase B over thirty minutes and holding at 98% mobile Phase B for 30 min to cover gradient delay.

A data-dependent acquisition method was used to analyze the nitro-BSA spike-in cell lysate digests where full survey scans were acquired from *m*/*z* 400–2000 at 240,000 mass resolution (at *m*/*z* 400). MS/MS spectra were acquired at 120,000 mass resolution on the top three most abundant ions with an isolation width of 5 using HCD (for MS/MS Scans 1 and 2) and CID (for MS/MS Scan 3). Dynamic exclusion was set for 30 s after one repeat count with a 500 exclusion list size. The extended isolation width was implemented to ensure co-isolation of precursor ions containing heavy or light nitrotyrosine. The resulting MS/MS spectra contained differential nitrotyrosine reporter ion peaks separated by 1 Da that were indicative of the relative amount of heavy or light nitropeptide.

Raw files were processed in MaxQuant 1.4.3 employing the Andromeda search algorithm using the Uniprot KB reference database for *Rattus norvegicus* [43]. A second database of known contaminants (that included BSA) provided with the MaxQuant suite was also employed. Constant modification of carbamidomethylation of cysteine and variable modifications of oxidized methionine, as well as light and heavy nitration of tyrosine were implemented in the search. A false discovery rate of 1% was used at both the peptide and protein level.

## 4. Conclusions

Stable isotope-labeled (^15^N) peroxynitrite was successfully synthesized to generate nitrated spike-in standards that were used to facilitate nitrotyrosine site identification and relative quantitation in a complex biological matrix. Based on the evaluation of nitrated peptide and protein standards reported here, we envision several applications for global-scale analysis of PTN using this approach. As an example, for *in vitro* applications, peroxynitrite (or other nitrating reagents) concentration or reaction time dependency can be assessed for multiple nitration targets in a complex protein mixture. For characterization of *in vivo* nitrative stress, relative quantitation is possible if appropriate ^15^N-labeled peroxynitrite concentrations are used and similar chemical selectivity is observed for the spike-in nitrated standard when compared to endogenous PTN. More importantly, the heavy nitrotyrosine reporter ion can be used as a diagnostic marker for the identification of endogenous nitration sites that can then be selected for further targeted validation if necessary. Future studies aim to use this approach to identify and quantify nitrotyrosine formation in various biological model systems of nitrative stress.

## Figures and Tables

**Table 1.** Relative quantitation of the bovine serum albumin (BSA) nitropeptide, Y*LYEIAR (*m*/*z* 487.2), identified from the microglial cell lysate digest background matrix. Ratio values are presented as the mean ± SD (indicating scan-to-scan variability). Individual technical replicates (*n* = 3) of the 0.25:1 ratio samples are shown as (**a**), (**b**) and (**c**).

Dilution	181/182	Normalized ratio
1:1	0.69 ± 0.02	1 ± 0.04
0.25:1 (**a**)	0.20 ± 0.04	0.28 ± 0.06
0.25:1 (**b**)	0.16 ± 0.01	0.23 ± 0.02
0.25:1 (**c**)	0.17± 0.01	0.24 ± 0.02
0.10:1	0.066 ± 0.004	0.094 ± 0.007

## References

[1] Abello N., Kerstjens H.A.M., Postma D.S., Bischoff R. (2009). Protein tyrosine nitration: Selectivity, physicochemical and biological consequences, denitration, and proteomics methods for the identification of tyrosine-nitrated proteins. J. Proteome Res.

[2] Anantharaman M., Tangpong J., Keller J.N., Murphy M.P., Markesbery W.R., Kiningham K.K., St. Clair D.K. (2006). B-amyloid mediated nitration of manganese superoxide dismutase: Implication for oxidative stress in a APP^NLh/NLh^ X PS-1^P264L/P264L^ double knock-in mouse model of Alzheimer’s disease. Am. J. Pathol.

[3] Aoyama K., Matsubara K., Fujikawa Y., Nagahiro Y., Shimizu K., Umegae N., Hayase N., Shiono H., Kobayashi S. (2000). Nitration of manganese superoxide dismutase in cerebrospinal fluids is a marker for peroxynitrite-mediated oxidative stress in neurodegenerative diseases. Ann. Neurol.

[4] Ckless K., Lampert A., Reiss J., Kasahara D., Poynter M.E., Irvin C.G., Lundblad L.K.A., Norton R., van der Vliet A., Janssen-Heininger Y.M.W. (2008). Inhibition of arginase activity enhances inflammation in mice with allergic airway disease, in association with increases in protein *S*-nitrosylation and tyrosine nitration. J. Immunol.

[5] Ito K., Hanazawa T., Tomita K., Barnes P.J., Adcock I.M. (2004). Oxidative stress reduces histone deacetylase 2 activity and enhances *IL-8* gene expression: Role of tyrosine nitration. Biochem. Biophys. Res. Commun.

[6] Neumann H., Hazen J.L., Weinstein J., Mehl R.A., Chin J.W. (2008). Genetically encoding protein oxidative damage. J. Am. Chem. Soc.

[7] Ohmori H., Kanayama N. (2005). Immunogenicity of an inflammation-associated product, tyrosine nitrated self-proteins. Autoimmun. Rev.

[8] Reynolds M.R., Reyes J.F., Fu Y., Bigio E.H., Guillozet-Bongaarts A.L., Berry R.W., Binder L.I. (2006). Tau nitration occurs at tyrosine 29 in the fibrillar lesions of Alzheimer’s disease and other tauopathies. J. Neurosci.

[9] Turko I.V., Li L., Aulak K.S., Stuehr D.J., Chang J.-Y., Murad F. (2003). Protein tyrosine nitration in the mitochondria from diabetic mouse heart: Implications to dysfunctional mitochondria in diabetes. J. Biol. Chem.

[10] Abriata L.A., Cassina A., Tórtora V., Marín M., Souza J.M., Castro L., Vila A.J., Radi R. (2009). Nitration of solvent-exposed tyrosine 74 on cytochrome c triggers heme iron-methionine 80 bond disruption: Nuclear magnetic resonance and optical spectroscopy studies. J. Biol. Chem.

[11] Aulak K.S., Koeck T., Crabb J.W., Stuehr D.J. (2003). Dynamics of protein nitration in cells and mitochondria. Am. J. Physiol.

[12] Koeck T., Fu X., Hazen S.L., Crabb J.W., Stuehr D.J., Aulak K.S. (2004). Rapid and selective oxygen-regulated protein tyrosine denitration and nitration in mitochondria. J. Biol. Chem.

[13] Khan J., Brennan D., Bradley N., Gao B., Bruckdorfer R., Jacobs M. (1998). 3-Nitrotyrosine in the proteins of human plasma determined by an ELISA method. Biochem. J.

[14] Shigenaga M.K., Lee H.H., Blount B.C., Christen S., Shigeno E.T., Yip H., Ames B.N. (1997). Inflammation and nox-induced nitration: Assay for 3-nitrotyrosine by HPLC with electrochemical detection. Proc. Natl. Acad. Sci. USA.

[15] Nikov G., Bhat V., Wishnok J.S., Tannenbaum S.R. (2003). Analysis of nitrated proteins by nitrotyrosine-specific affinity probes and mass spectrometry. Anal. Biochem.

[16] Prokai L., Guo J., Prokai-Tatrai K. (2014). Selective chemoprecipitation to enrich nitropeptides from complex proteomes for mass-spectrometric analysis. Nat. Protoc.

[17] Prokai-Tatrai K., Guo J., Prokai L. (2011). Selective chemoprecipitation and subsequent release of tagged species for the analysis of nitropeptides by liquid chromatography—Tandem mass spectrometry. Mol. Cell. Proteomics.

[18] Mann M., Jensen O.N. (2003). Proteomic analysis of post-translational modifications. Nat. Biotechnol.

[19] Nørregaard Jensen O. (2004). Modifi qacation-specific proteomics: Characterization of post-translational modifications by mass spectrometry. Curr. Opin. Chem. Biol.

[20] Petersson A.S., Steen H., Kalume D.E., Caidahl K., Roepstorff P. (2001). Investigation of tyrosine nitration in proteins by mass spectrometry. J. Mass Spectrom.

[21] Falick A., Hines W., Medzihradszky K., Baldwin M., Gibson B. (1993). Low-mass ions produced from peptides by high-energy collision-induced dissociation in tandem mass spectrometry. J. Am. Soc. Mass Spectrom.

[22] Papayannopoulos I.A. (1995). The interpretation of collision-induced dissociation tandem mass spectra of peptides. Mass Spectrom. Rev.

[23] Hohmann L.J., Eng J.K., Gemmill A., Klimek J., Vitek O., Reid G.E., Martin D.B. (2008). Quantification of the compositional information provided by immonium ions on a quadrupole-time-of-flight mass spectrometer. Anal. Chem.

[24] Steen H., Küster B., Fernandez M., Pandey A., Mann M. (2001). Detection of tyrosine phosphorylated peptides by precursor ion scanning quadrupole tof mass spectrometry in positive ion mode. Anal. Chem.

[25] Bateman R., Carruthers R., Hoyes J., Jones C., Langridge J., Millar A., Vissers J. (2002). A novel precursor ion discovery method on a hybrid quadrupole orthogonal acceleration time-of-flight (Q-TOF) mass spectrometer for studying protein phosphorylation. J. Am. Soc. Mass Spectrom.

[26] Chiappetta G., Corbo C., Palmese A., Marino G., Amoresano A. (2009). Quantitative identification of protein nitration sites. Proteomics.

[27] Guo J., Prokai-Tatrai K., Prokai L. (2012). Relative quantitation of protein nitration by liquid chromatography-mass spectrometry using isotope-coded dimethyl labeling and chemoprecipitation. J. Chromatogr.

[28] Colzani M., Schütz F., Potts A., Waridel P., Quadroni M. (2008). Relative protein quantification by isobaric silac with immonium ion splitting (ISIS). Mol. Cell. Proteomics.

[29] Zhang G., Neubert T.A. (2006). Automated comparative proteomics based on multiplex tandem mass spectrometry and stable isotope labeling. Mol. Cell. Proteomics.

[30] Robinson K.M., Beckman J.S. (2005). Synthesis of peroxynitrite from nitrite and hydrogen peroxide. Methods Enzymol.

[31] Hebert A.S., Merrill A.E., Bailey D.J., Still A.J., Westphall M.S., Strieter E.R., Pagliarini D.J., Coon J.J. (2013). Neutron-encoded mass signatures for multiplexed proteome quantification. Nat. Methods.

[32] Bell-Temin H., Barber D.S., Zhang P., Liu B., Stevens S.M. (2012). Proteomic analysis of rat microglia establishes a high - confidence reference data set of over 3000 proteins. Proteomics.

[33] Bell-Temin H., Zhang P., Chaput D., King M.A., You M., Liu B., Stevens S.M. (2013). Quantitative proteomic characterization of ethanol-responsive pathways in rat microglial cells. J. Proteome Res.

[34] Cox J., Mann M. (2008). Maxquant enables high peptide identification rates, individualized ppb-range mass accuracies and proteome-wide protein quantification. Nat. Biotechnol.

[35] Seeley K.W., Stevens S.M. (2012). Investigation of local primary structure effects on peroxynitrite-mediated tyrosine nitration using targeted mass spectrometry. J. Proteomics.

[36] Stevens S.M., Prokai-Tatrai K., Prokai L. (2008). Factors that contribute to the misidentification of tyrosine nitration by shotgun proteomics. Mol. Cell. Proteomics.

[37] Rose C.M., Merrill A.E., Bailey D.J., Hebert A.S., Westphall M.S., Coon J.J. (2013). Neutron encoded labeling for peptide identification. Anal. Chem.

[38] Cook S., Jackson G.P. (2011). Characterization of tyrosine nitration and cysteine nitrosylation modifications by metastable atom-activation dissociation mass spectrometry. J. Am. Soc. Mass Spectrom.

[39] Jones A., Mikhailov V., Iniesta J., Cooper H. (2010). Electron capture dissociation mass spectrometry of tyrosine nitrated peptides. J. Am. Soc. Mass Spectrom.

[40] Mikhailov V.A., Iniesta J., Cooper H.J. (2010). Top-down mass analysis of protein tyrosine nitration: Comparison of electron capture dissociation with “slow-heating” tandem mass spectrometry methods. Anal. Chem.

[41] Hughes M.N., Nicklin H.G. (1968). The chemistry of pernitrites. Part I. Kinetics of decomposition of pernitrous acid. J. Chem. Soc.

[42] Wisniewski J.R., Zougman A., Nagaraj N., Mann M. (2009). Universal sample preparation method for proteome analysis. Nat. Methods.

[43] UniProt Knowledgebase http://www.uniprot.org/uniprot/?query=organism:10116+keyword:1185.

